# Editorial: Epidemiology and clinical researches on neuropsychiatric disorders in aging

**DOI:** 10.3389/fpsyt.2023.1108474

**Published:** 2023-01-18

**Authors:** Wuxiang Xie, Baoliang Zhong, Lirong Liang, Yutong Samuel Cai

**Affiliations:** ^1^Peking University Clinical Research Institute, Peking University First Hospital, Beijing, China; ^2^Department of Psychiatry, Wuhan Mental Health Center, Wuhan, China; ^3^Department of Clinical Epidemiology, Beijing Institute of Respiratory Medicine and Beijing Chao-Yang Hospital, Capital Medical University, Beijing, China; ^4^Centre for Environmental Health and Sustainability, University of Leicester, Leicester, United Kingdom

**Keywords:** aging, depression, cognitive function, epidemiology, clinical research

With the rising aging population in a global range, related neuropsychiatric disorders such as depression and dementia, have emerged and caused a tremendous disease burden. Over the past decades, many risk factors have been identified (1–12), and advances have been made in developing prevention and intervention strategies. However, there still exist challenges to be addressed. These challenges include but are not limited to early detection and prediction of neuropsychiatric disorders, comorbidities of both neuropsychiatric and non-neuropsychiatric aspects, identifying novel indicators for disease progression and prognosis, as well as investigating potential mediating mechanisms. Facing unprecedented challenges, we launched this Research Topic to promote healthy aging and longevity from the neuropsychiatric perspective, *via* collaboration from a number of professional disciplines.

In this topic, Qiu et al. conducted a pooled analysis of two national aging cohort studies to explore a bidirectional relationship between body pain and depressive symptoms. This study detected bidirectional longitudinal influence, while most of the previous studies only explored a cross-sectional or unidirectional longitudinal association. The results indicated that medical practitioners should be aware of the bidirectional relationship and carry out early assessments or event interventions to reduce the possibility of depression in people with pain or to prevent pain in people with depressive symptoms.

Liu et al. performed a resting-state functional MRI study to investigate the altered intrinsic brain activity (IBA) in patients suffering from late-life depression (LLD) using a percent amplitude of fluctuation (PerAF) method. This study mainly found that compared with the normal control group, the late-life depression (LLD) group demonstrated decreased PerAF differences in the bilateral superior frontal gyrus, orbital part (Frontal_Sup_Orb), and bilateral anterior cingulate cortex (ACC), which was closely related to their attention control defects.

In a cross-sectional study published in this topic, Pan Y. et al. identified an association between poor socioeconomic status (SES) and increased prevalence of dependency personality disorder (DPD), and the results of this work represent preliminary evidence that perceived social status has a stronger association with DPD than objective SES. The impact of subjective SES on DPD is possibly associated with the perception of position in the social hierarchy. However, there is abundant room for further progress in determining the pathways of subjective SES and DPD to fully understand the complicated causality and provide targeted support strategies to reduce or delay the dependency of elderly people.

Pan W. et al. conducted a pilot study to identify cognitive function processes that may be associated with treatment response in LLD. In this study, first-episode LLD patients treated with 8-week of escitalopram or sertraline demonstrated improvement of depression and partial cognitive function including immediate memory, language, and delayed memory. The preliminary findings suggested that working memory, attention, visuospatial, and language function should be examined further as a means of providing the useful objective biomarkers of treatment response.

In a longitudinal cohort study, Han et al. examined the relationship between depressive symptoms and the changes in serum cystatin C levels, and they found that baseline persistent depressive symptoms were significantly associated with an increased rate of serum cystatin C levels during the 10-year follow-up. This study indicated that the level of serum cystatin C should be monitored in the elderly with persistent depressive symptoms. Potential mechanisms of the relationship between kidney dysfunction and depression need to be further characterized.

In another large-scale longitudinal cohort study, Wang et al. found that sustained or increasing physical activity was linked to slower declines in motor function and lower risk of incident frailty. These findings are encouraging, not confined to participants with persistently active participation in physical activity, who can still gain substantial benefits in the quality of life by becoming more physically active irrespective of past physical activity levels, providing further evidence to the broad public health benefits of physical activity.

Xiao and Huang performed a cross-sectional study based on the data from the National Health and Nutrition Examination Survey, and found that high levels of dietary inflammatory index were associated with depression and suicidal ideation in older adults. In another cross-sectional study among 769 elderly primary care attenders from 13 primary care clinics in Wuhan, Zhu et al. reported that 12-month prevalence of suicidal ideation was 16.6%, which indicates the potentially high and urgent needs for suicide prevention and crisis intervention in Chinese older adults receiving primary care. Due to the rapid social changes and accelerating aging in recent decades, mental health problems and suicidal behaviors have been significant public health challenges for the older adults (13–17). Recognizing older adults with suicidal ideation in primary care settings could facilitate the early prevention of suicide in the elderly population.

In the last article published in this topic, Feng et al. presented the 17-year temporal trend in anxiety and/or depression prevalence in patients hospitalized for acute exacerbation of chronic obstructive pulmonary disease (AECOPD) in Beijing, which included 382,125 records from a city-wide representative database of hospitalizations. Three segments in the temporal trend were observed, with a mild increase during 2004–2009 (from 0.4 to 0.6%), followed by a sharp increase during 2009–2012 (from 0.6 to 2.5%), then stabilized at about 3% during 2012–2020. They also reported that patients with anxiety and/or depression had longer hospital stays, more medical costs and higher risks of readmission for AECOPD. These results show that anxiety and/or depression are undoubtedly important burdens for people with AECOPD, the health care systems and the medical insurance systems. Providing appropriate diagnosis and effective treatment of anxiety and/or depression for patients with AECOPD needs to be strengthened in clinical practice.

To conclude, the articles published in this topic provide additional evidence from epidemiology and clinical researches for current literature on neuropsychiatric disorders in aging. In the future, high-quality epidemiology and clinical researches, especially randomized controlled trials are needed to develop effective strategies of prevention and intervention for neuropsychiatric disorders in aging.

## Author contributions

WX writing original draft of this editorial. All authors contributed to this topic design and manuscript editing efforts. All authors contributed to the article and approved the submitted version.
